# Adverse childhood experiences and maternal anxiety and depression: a meta-analysis

**DOI:** 10.1186/s12888-020-03017-w

**Published:** 2021-01-11

**Authors:** Nicole Racine, Chloe Devereaux, Jessica E. Cooke, Rachel Eirich, Jenney Zhu, Sheri Madigan

**Affiliations:** 1 Department of Psychology, Faculty of Arts, University of Calgary, 2500 University Dr. NW., Calgary, AB T2N 1N4 Canada; 2 Alberta Children’s Hospital Research Institute, Calgary, AB T2N 1N4 Canada

**Keywords:** Adverse childhood experiences, Anxiety, Depression, Pregnancy, Postpartum

## Abstract

**Background:**

It has been proposed that adverse childhood experiences (ACEs) can put women at risk for mental illness in the pregnancy and postpartum periods. While some studies have found strong support for this proposition, others have found weak or no support. This study is a meta-analysis of the association between ACEs and maternal mental health to resolve between-study discrepancies, and to examine potential moderators of associations.

**Methods:**

Three electronic databases (i.e., MEDLINE, Embase, and PsycINFO) were searched up to November 2018 by a health sciences librarian. A hand search was conducted in January 2020 and relevant studies were added. Included studies reported on associations between ACEs and maternal depression and/or anxiety in the perinatal period (pregnancy to 1-year postpartum). Pregnancy and postpartum outcomes were examined separately for both depression and anxiety. Random-effect meta-analyses were conducted. Moderator analyses were conducted using meta-regression. Study quality was evaluated using a 15-point scale.

**Results:**

The initial search yielded 4646 non-duplicate records and full text review occurred for 196 articles. A total of 15 studies (*N* = 7788) were included in the meta-analyses, of which 2 were also described narratively. Publication year ranged from 1998 to 2019. Mothers were approximately 28.93 years of age when they retrospectively reported on their ACEs. All studies had maternal self-report questionnaires for the mental health outcomes. Study quality ranged from 7 to 12. The pooled effect sizes between ACEs and prenatal (*N* = 12; *r* = .19; 95% CI= .13, .24) and postpartum (*N* = 7; *r* = .23; 95% CI = .06 to .39) depressive symptoms were significant. The pooled effect size between ACEs and prenatal anxiety was also significant (*N* = 5; *r* = .14; 95% CI= .07, .21). Moderator analyses indicated that timing of depressive and anxiety symptoms may be important for understanding associations.

**Conclusions:**

ACEs confer risk to maternal mental health, albeit effect sizes are small to moderate in magnitude. Trauma-informed approaches, as well as increased mental health support during and after pregnancy, may help to offset the relative risk of ACEs on maternal mental health.

**Supplementary Information:**

The online version contains supplementary material available at 10.1186/s12888-020-03017-w.

## Background

It is well established that maternal mental health difficulties are common. Approximately 20–30% of women in low- and middle-income countries and 10% of women in high-income countries have a mental illness, such as anxiety or depression, during pregnancy or in the postpartum period [1, 2]. Maternal mental health difficulties are particularly problematic because they put both maternal and child health at risk [3]. For example, pregnant women with mental health difficulties are more likely to have poor health, such as obstetrical complications and preterm labour [4], and to maintain health-risk behaviours such as alcohol and drug use in pregnancy [5]. In addition maternal mental health difficulties are associated with increased risk for poor infant developmental health (i.e., physical, cognitive, socio-emotional) [6–9]. A recent prevalence study in the UK found that anxiety and depression are the most common maternal mental illnesses and that 53% of children are at risk of being exposed to maternal mental illness prior to age 16 years [10]. Thus, understanding and mitigating the precursors to maternal mental illness in pregnancy and after childbirth are critical for reducing this large-scale public health burden.

Maternal mental health difficulties are strongly associated with social determinants of health such as poverty, devalued social roles, and gender-based violence [11]. Experiences of trauma and violence also put women disproportionately at risk for mental health difficulties in the perinatal period. An emerging body of research has examined how adverse childhood experiences (ACEs), such as abuse, neglect, and household dysfunction experienced prior to the age of 18, have downstream consequences on mental health in the perinatal period (i.e., pregnancy and postpartum) (e.g., [12, 13]). It has been theorized that experiences of adversity early in life may make an individual more vulnerable to experiencing mental health difficulties in adulthood via alteration to the stress response and affect regulation systems [14], which play a key role in the development of mental health difficulties [15]. Indeed, previous research has demonstrated that the accumulation of ACEs puts women at risk for depressive symptoms both in pregnancy and the postpartum period [12, 13]. As a result of this body of research, there has been a call to implement trauma-informed approaches to perinatal care [16, 17]. To inform trauma-informed approaches to patient care, it is critical to evaluate the state of the science on ACEs and maternal mental health outcomes.

Despite several studies demonstrating an association between exposure to ACEs and poor maternal mental health, there is also research that has found no association between exposure to ACEs and maternal postpartum depression [18]. Given these conflicting findings, the first goal of the current study is to conduct a meta-analysis of the association between ACEs and maternal mental health difficulties in pregnancy and the postpartum period. When study findings are heterogeneous, it often suggests that there are important moderators of the association. That is, there are methodological or social characteristics that may amplify or attenuate associations. For example, previous research suggests there are different clinical profiles of perinatal depression and anxiety based on timing of symptom onset, such that earlier onset of symptoms in pregnancy and postpartum may be indicative of more severe and chronic problems [19]. Thus, examining the timing of perinatal mental health difficulties in relation to maternal ACE exposure may allow for more tailored screening and treatment during the perinatal period [19]. Further methodological differences, such as study quality, may help to explain heterogeneous findings. Additionally, sociodemographic risk (e.g., low income) may moderate the association by exerting an additive risk effect on the link between ACEs and maternal mental health [20, 21]. Finally, exposure to an increased number of ACEs may confer a greater risk of clinically severe perinatal depression and/or anxiety [22]. Thus, the second goal of the current study is to identify moderators of the association between ACEs and maternal mental health in order to explain “when” and “for whom” associations are stronger or weaker.

In sum, in order to elucidate health disparities in maternal mental health research, the current meta-analytic study had two aims: 1) provide a pooled effect size of the association between ACEs and maternal mental health difficulties (i.e., anxiety and depression) in pregnancy and the postpartum periods among observational studies, and 2) to examine potential moderators of this association.

## Methods

### Definitions of constructs

*Adverse Childhood Experiences* (ACEs) included cumulative retrospective self-reports of child adversity prior to the age of 18 years, including maltreatment and household dysfunction. Measures that were included either consisted of the original 8-item ACEs developed by Felitti and colleagues [23], including physical abuse, sexual abuse, emotional abuse, parent substance use, parent divorce/separation, parent incarceration, parent mental health problem, and exposure to domestic violence, or the 10-item version, which includes the addition of physical neglect and emotional neglect. *Prenatal depressive symptoms* included depressive symptoms either self-reported (e.g., Center for Epidemiologic Studies Depression Scale) or assessed (e.g., diagnostic codes or diagnosed depressive disorder) during pregnancy. *Postpartum depressive symptoms* included depressive symptoms either self-reported (e.g., Edinburgh Postnatal Depression Scale) or assessed (e.g., diagnosis of depression) after the birth of a child and prior to 12-months postpartum. *Prenatal anxiety symptoms* included anxiety symptoms either self-reported (e.g., State Trait Anxiety Scale) or assessed (e.g., diagnosed anxiety disorder) during pregnancy. *Postpartum anxiety symptoms* included those either self-reported (e.g., Generalized Anxiety Disorder, 7-item) or assessed (e.g., diagnosed disorder) after the birth of a child and prior to 12-months postpartum. *Perinatal depression* and *perinatal anxiety* referred to measurements of anxiety and depression that occurred in both pregnancy and the postpartum period.

### Search strategy

PRISMA guidelines were used for this meta-analysis [24]. The PRISMA checklist is included in Supporting Information 1. Searches were conducted in MEDLINE, Embase, and PsycINFO up to November 2018 by a health sciences librarian. A hand search was conducted in January 2020 and relevant studies were added. This meta-analysis was part of a broader search on adverse childhood experiences (ACEs) and thus outcome terms were not restricted or included as search terms (See Supporting Information 2). Text word fields were searched for the acronym ACEs along with the phrase “adverse childhood event or experiences”. Both adjacency operators and truncation symbols were used to capture variations in phrasing. Years were limited from 1998 when the original ACEs study occurred [23]. No language restrictions were applied.

### Study inclusion and exclusion criteria

**Fig. 1:**
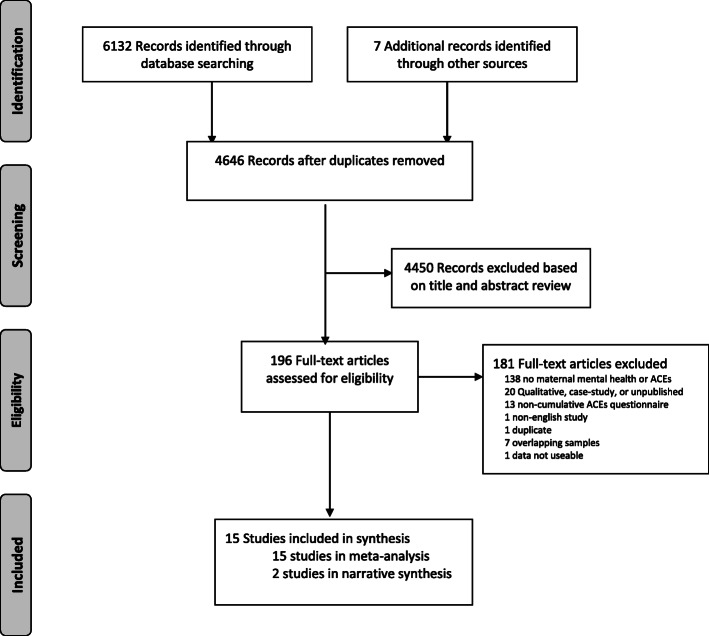
PRISMA flow diagram detailing the search strategy

Titles and abstracts yielded from the search strategy were reviewed by 2 independent coders and evaluated to determine inclusion criteria, which were as follows: (1) ACEs prior to the age of 18 years; (2) a measure of maternal depression or anxiety in pregnancy or prior to 12-months postpartum; and (3) statistical data that could be transformed into an effect size. As outlined in Fig. 1, full-length studies were excluded if they (1) did not have a maternal mental health or ACEs measure; (2) were qualitative in nature; (3) did not have a cumulative ACEs measure; (4) were not in English; (5) were from an overlapping sample, or (6) an effect size could not be extracted.

### Study quality assessment

A 15-point scale was used to evaluate the quality of included studies based on a tool adapted from the National Institutes of Health (NIH) Quality Assessment Tool for Observational Cohort and Cross-sectional studies (See Supporting Information 3). Study quality was assessed by one primary coder and 20% of the ratings were verified by a second coder. The intraclass correlation among coders using a random effects model was .81. Discrepancies were resolved through consensus. All ratings are provided in Supporting Information 4.

### Data extraction

Effect sizes for the association between maternal ACEs and mental health outcomes were extracted. Moderator variables were also extracted including: (1) syndromal level of mental health difficulty (i.e., categorized as 1 = majority of sample had a diagnosed mental health disorder, 0 = majority of sample did not have a diagnosed mental health disorder); (2) sociodemographic risk (i.e., 1 = sample had one or more of the following risks: low income, single parents, low education, adolescents, mostly racial and/or ethnic minorities, or pre-existing health difficulties, 0 = no sociodemographic risk); (3) maternal age at mental health measurement; (4) prenatal (i.e., weeks of pregnancy) or postpartum time (i.e., months post-partum) when outcome measured; and (5) study quality, based on NIH tool described above. All studies meeting inclusion criteria were independently coded by 2 trained coders. Reliability for continuous moderators ranged from 80.00 to 100.00% agreement and for categorical moderators the mean percentage agreement was 86.70%. Discrepancies were resolved through consensus.

### Data synthesis

Effect sizes, including correlation coefficients, regression coefficients, chi-square values, odds-ratios, and medians were extracted from studies. While the majority of studies reported raw correlation coefficients, all other measures of effect size were converted to correlations for ease of analysis and interpretation. As not all studies adjusted for the same covariates, the use of unadjusted statistics was employed [25].

Only one effect size per study was selected. If there were multiple studies based on the same dataset, the study with the largest sample size and most complete data extraction information was selected. If a study assessed both depression and anxiety, as well as provided different time points of measures (i.e. prenatal and postpartum), all effect sizes were extracted and included in separate meta-analyses. If multiple measurements of anxiety or depression were provided (e.g., Center for Epidemiological Studies Depression Scale and Patient Health Questionnaire), a pooled effect size was derived and used in the meta-analysis.

### Data analysis

Meta-analyses were conducted when there were more than three studies; otherwise findings were described narratively. Pooled effect sizes and moderator analyses were conducted using Comprehensive Meta-Analysis Software, Version 3.0 [26]. When a study reported non-significant findings without an effect size (*n* = 1), a *p*-value of .50 was entered, consistent with recommendations by Rosenthal [27]. Pooled effect sizes are represented as correlations *r* with 95% confidence intervals. Using guidelines provided by Funder and Ozer [28], correlations of .10, .20, and .30 were considered small, medium, and large in magnitude, respectively. All calculations were based on random-effects models given the assumption of variation in population parameters among studies [29]. Outliers were examined by investigating whether the confidence intervals of studies overlapped with the overall effect [30]. Only one study did not overlap with the confidence intervals [12]. Sensitivity analyses determined that exclusion of this study did not significantly impact the overall effect size (*r* = .199, 95% CI = .15, .25) and thus the study was retained. Sensitivity analyses based on outliers, attrition and study quality were also conducted.

Heterogeneity of effect sizes was examined using the *Q* and *I*^*2*^ statistics. Examination of moderators is warranted when the *Q* statistic is significant and/or when the *I*^*2*^ statistic is greater than 50% [29]. Significance of categorical and continuous moderators was determined by the *Q* statistic and by meta-regressions, respectively [31]. Based on recommendations from Borenstein and colleagues [29], categorical moderators were only explored when more than 3 studies per level of the moderator were available. The Egger test and examination of funnel plots were examined to explore potential publication bias.

## Results

### Studies selected

Details of the process for study selection can be found in the PRISMA diagram (Fig. 1). The initial search yielded 4646 non-duplicate records and full text review occurred for 196 articles. A total of 15 studies are included in the meta-analyses [12, 13, 18, 22, 32–42], of which 2 are also described narratively [18, 37]. Characteristics of the included studies can be found in Table 1.

**Table 1:** Characteristics of studies included

First Author, Year, Reference	N^a^	Mat age at ACEs measure (years)	Country	Mat mental health measure	Mat mental health time point	Name of mat mental health measure	Type of ACEs Measure
Angerud 2018 [22]	1188	30.83	Sweden	Depression	Prenatal Postpartum	EPDS	10-item
Appleton 2019 [32]	126	28.9	USA	Depression	Prenatal	EPDS	10-item
Folger 2018 [33]	257	–	USA	Depression	Postpartum	EPDS	10-item
Fredriksen 2017 [34]	762	30.3	Norway	Depression Anxiety	Prenatal Postpartum	EPDS PRAQ-R	10-item
Hantsoo 2019 [35]	48	27.97	USA	Depression Anxiety	Prenatal	EPDS STAI State STAI Trait	10-item
Howell 2017 [36]	101	26	USA	Depression	Prenatal	CES-D	10-item
Letourneau 2019 [37]	907	32.16	Canada	Depression Anxiety	Prenatal Postpartum	EDS SCL-90-R– Anxiety	10-item
Menke 2018 [18]	250-prenatal 328- postpartum	–	USA	Depression Anxiety	Prenatal Postpartum	EPDS-10 GAD-7	10-item
Mersky 2018 [38]	735	23.7	USA	Depression	Prenatal	EPDS	10-item
Miller-Graff 2018 [39]	101	26	USA	Depression	Prenatal	CES-D	10-item
Morrison 2017 [40]	25	26.92	USA	Depression	Postpartum	EPDS	10-item
Narayan 2018 [41]	101	29.1	USA	Depression	Prenatal	EPDS	10-item
Racine 2020 [12]	1994	33.87	Canada	Depression	Prenatal Postpartum	EPDS	8-item
Wajid 2019 [13]	636	–	Canada	Depression	Prenatal	EPDS	10-item
Young-Wolff 2019 [42]	355	30	USA	Depression Anxiety	Prenatal	PHD-9	8-item

*CES-D* Centre for Epidemiologic Studies Depression Scale, *EDS* Edinburgh Depression Scale, *EPDS* Edinburgh Postpartum Depression Scale, *GAD-7* Generalized Anxiety Disorder 7-Item, *PHQ-9* Patient Health Questionnaire, *PRAQ-R* Pregnancy-Related Anxiety Questionnaire-Revised, *SCL-90-R* Symptom Checklist-90, Revised, *STAI State* State Trait Anxiety Inventory- State, *STAI Trait* State Trait Anxiety Inventory- Trait. -: value not reported in the manuscript

^a^Sample size entered into the meta-analysis

### Sample characteristics

Of the studies included in the meta-analyses, the total number of participants was 7788 with samples sizes ranging from 25 to 1994. Publication year ranged from 1998 to 2020. In all studies, mothers provided a retrospective report of their ACEs. On average, mothers were 28.81 years of age (range 23.70 to 33.87) when they retrospectively reported on their ACEs. The majority of studies used the 10-item ACEs questionnaire (*n* = 13) with only 2 studies using the 8-item questionnaire. All studies had maternal report questionnaires for the mental health outcomes. There were a total of *n* = 12 studies with prenatal depression outcomes, *n* = 7 studies with postpartum depression outcomes, *n* = 5 studies with prenatal anxiety outcomes, and *n* = 2 studies with postpartum anxiety outcomes. Two studies had depression outcomes measured in pregnancy or the postpartum period (i.e., perinatal depression) and one study had perinatal anxiety outcomes. Only one study included a clinical sample and 6 studies used cross-sectional designs while the other 9 studies had longitudinal designs. Of studies included in the meta-analysis, 66.67% (*n* = 10) were conducted in the USA, 20.00% in Europe (*n* = 3), and 13.30% in Canada (*n* = 2). Study quality ranged from 7 to 12 (Mean= 9.07, SD=1.5). Only one study few within the range of low methodological quality, defined as 1 SD below the mean.

### Meta-analytic results for maternal ACEs and prenatal depressive symptoms

**Fig. 2:**
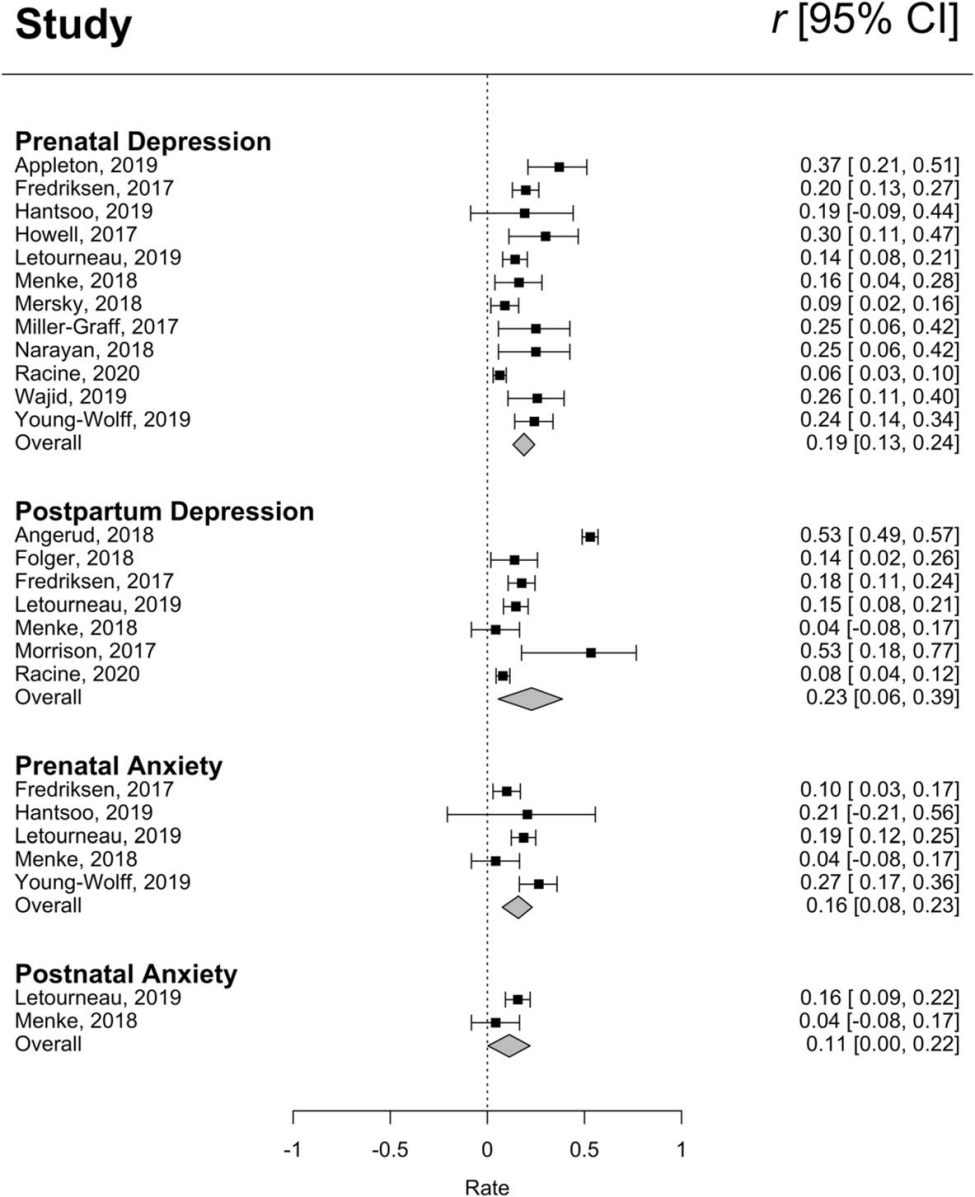
Forest plot of effect sizes for maternal ACEs and mental health in pregnancy and the postpartum

A total of 12 studies (6116 participants) were available for this random-effects meta-analysis, which produced a significant and positive pooled effect size *r* = .19 (95% CI = .13, .24; Fig. 2). Thus, a higher number of maternal ACEs was associated with higher reports of depressive symptoms in pregnancy. The funnel plot inspection revealed asymmetry (Supporting Information 5) and the Egger test was significant, indicating that studies with smaller sample sizes had more extreme values. When results were adjusted by adding 5 imputed studies using Duval and Tweedie’s trim and fill technique, the adjusted effect size was *r* = .15 (95% CI =.09, .20; Fig. 2), but remained statistically significant. Between study heterogeneity was indicated (*Q* = 42.38, *p* <.001 and *I*^2^ =74.05) and moderators were explored (Supporting Information 6); however, none emerged as significant.

Sensitivity analyses that removed one study that had the highest level of attrition [12] was conducted, however the effect size without this study (*r* = .199, 95% CI =.15, .25) did not significantly differ from the overall pooled effect size. Sensitivity analyses that excluded the lower quality study were also conducted [36], however no significant difference emerged (*r* = .17, 95% CI = .11, .23).

### Meta-analytic results for maternal ACEs and postpartum depressive symptoms

A total of 7 studies (5461 participants) were available for this random-effects meta-analysis, which produced a significant and positive pooled effect size of *r* = .23 (95% CI = .06, .39; Fig. 2). Thus, a higher number of maternal ACEs was associated with higher reports of postpartum depressive symptoms. The funnel plot inspection was symmetrical (See Supporting Information 7) and the Egger’s test was not significant (*p* = .73), indicating no apparent publication bias. Between study heterogeneity was indicated (*Q* = 235.95, *p* <.001 and *I*^2^ = 97.46) and moderators were explored (Supporting Information 8). One significant moderator emerged: the association between postpartum depression and maternal ACEs was stronger when assessed at later postpartum timepoint. Specifically, for every 2 month unit increase in the postpartum period, the effect size increased by .04 (95% CI: .01, .08).

A sensitivity analysis was conducted without one study with a small effect size (*n* = 25) [40]. The overall pooled effect size when this study was excluded did not significantly differ from the overall pooled effect size reported (*r* = .196, 95% CI = 0.15, .37). Another sensitivity analysis examined whether excluding a study with a higher attrition level [12] would impact the pooled effect size, however there was no significant difference (*r* = .256, 95% CI = .06, .44), as demonstrated by the overlapping confidence intervals.

### Meta-analytic results for maternal ACEs and prenatal anxiety

A total of 5 studies (2322 participants) were available for this random-effects meta-analysis, which produced a significant and positive pooled effect size of *r* = .14 (95% CI =.07, .21; Fig. 2). Thus, a higher number of maternal ACEs was associated with higher reports of prenatal anxiety symptoms. The funnel plot inspection was symmetrical (see Supporting Information 9) and the Egger’s test was not significant (*p* = .99). Between study heterogeneity was indicated (*Q* =11.27, *p* = .02 and *I*^2^ = 64.49) and all moderators are reported in Supporting Information 10. One significant moderator emerged: the association between maternal ACEs and prenatal anxiety was highest when measured earlier in pregnancy as compared to later in pregnancy. Across studies, timing of the prenatal anxiety symptom measurement ranged from 18 to 23 weeks gestation. The meta-regression results revealed that for every 1 week unit increase in pregnancy across this range, the effect size decreased by .06 (95% CI = −.11, −.01).

### Narrative results for maternal ACEs and postpartum anxiety

Two studies with a total of 1235 participants examined the association between maternal ACEs and anxiety in postpartum women [18, 37]. The first study [37] found a small significant association between maternal ACEs and postpartum anxiety (*b* = .16, *p* <.05), while the second study [18] did not find a significant association.

## Discussion

The current study indicates that maternal ACEs are positively associated with both prenatal and postpartum depression (*r* = .19 and *r* = .23, respectively), as well as prenatal anxiety (*r* = .14). Overall, these results are consistent with the view that maternal ACEs are an important risk factor for poor mental health in pregnancy and postpartum period. Several processes have been proposed to explain how maternal ACEs confer a significant risk for mental health. For example, Danese and McEwen [43] describe how childhood adversity contributes to allostatic load, or wear-and-tear on the body, resulting in physiological changes to the nervous, endocrine, and immune systems. These biologically embedded changes have been hypothesized to result in greater susceptibility to mental illness when faced with psychosocial stressors [44], especially during pregnancy, when mothers face substantial neuroendocrinological [45] and psychosocial [46, 47] changes. Specifically, memories and cognitions regarding childhood abuse and household dysfunction may be particularly salient sources of stress during the pregnancy and transition to parenthood periods. Thus, identifying and increasing buffers of psychosocial stress (e.g., social support [48];) may be effective strategies to reduce the impact of ACEs on depression during the perinatal period.

The association between maternal ACEs and postpartum depression was stronger when measured later (up to 12 months) versus earlier in the postpartum period. Previous literature has found that postpartum depression tends to occur approximately 4 weeks after delivery [49]. However, for some women, postpartum depression may persist well beyond this timeframe and become chronic [50]. Our findings suggest that ACEs may put mothers at risk for depression across the perinatal period, which has been demonstrated to have particularly deleterious consequences for child and family outcomes [51, 52]. More research is needed to better understand the course, continuity, and mechanisms of postpartum depression in women with ACEs, as well as interventions to offset its potential chronicity. Clinicians should be aware of the possible vulnerability to chronic depression in mothers with ACEs and monitor women at risk for continued symptoms throughout the postpartum period and beyond to offset potential disease burden.

The results also suggest that exposure to childhood adversity may confer a risk of developing prenatal anxiety. Adverse childhood experiences may be associated with particular physiological changes and health risks that increase the susceptibility to anxiety [45, 53, 54]. While a paucity of research examining maternal ACEs and postnatal anxiety exists, studies have shown detrimental impacts of prenatal anxiety on child development. Specifically, prenatal anxiety may increase the risk for child socioemotional problems [8] and poorer cognitive outcomes [7]. Thus, it remains critical to examine the impact of childhood adversity on maternal mental health. Finally, moderator analyses revealed that associations among ACEs and prenatal anxiety were strongest in the first weeks of pregnancy. Thus, early identification and monitoring for anxiety in pregnancy, particularly for mothers with ACEs, may best allow for intervention to curtail future development concerns in their children.

Research on ACEs in primary care settings have signaled that the majority of women (71.2%) report at least one childhood adversity, while a smaller number (20.5%) report experiencing four or more of these adversities [55]. In the prenatal primary care setting, at least 46% of women report experiencing at least one childhood adversity [56]. Given the high prevalence of childhood adversity in the general population, as well as the demonstrated association between ACEs and maternal mental health identified in the current study, trauma informed approaches to prenatal and postpartum care are warranted. Trauma-informed approaches involve being informed about the impact of trauma on health and mental health, being compassionate in providing care, and having procedures and policies that resist causing distress to individuals who may have a trauma history. In some instances, asking about an individual’s trauma history may be relevant for their medical care, but in other instances it may not [17]. Prioritizing trauma-informed approaches to care is more important than simply providing an ACEs questionnaire to ask about childhood adversity. It is also critical for healthcare providers to consider cumulative adversity that may have occurred across the lifecourse and how this may influence maternal mental health.

### Limitations

Several limitations of the current meta-analytic study should be noted. First, assessment of maternal ACEs was based on retrospective self-report. Recall of ACEs history may be susceptible to memory inaccuracies which may be especially compromised among participants who have experienced trauma [57]. We did not include other retrospective measures of child maltreatment or adversity in the current study in order to obtain more homogeneous estimates of the association between the cumulative nature of ACEs and maternal mental health. Future meta-analyses may consider broadening the search criteria to include discrete adversity items (e.g., physical abuse or sexual abuse), as well as other measures of childhood maltreatment and trauma. Second, analyses are based on reports of depressive and anxiety symptoms, not diagnoses of disorders. It will be important in future research to determine if maternal ACEs increase risk for mood or anxiety disorders in the perinatal period. Third, the present study could only narratively describe the association between maternal ACEs and postpartum anxiety due to a small number of available studies. This represents an important area for future research given the heterogeneous findings of the presently included studies. Fourth, no protocol was registered for the current review as it was part of a large series of meta-analyses that were conducted on ACEs. Finally, the majority of participants in the included studies resided in North America, and as such, these results may be limited in terms of their generalizability across cultures and countries outside of North America.

## Conclusions

In sum, this study sheds light on the associations between maternal ACEs and subsequent mental health difficulties during pregnancy and the postpartum period. Specifically, our findings suggest that ACEs are likely an important determinant of maternal mental health difficulties, particularly prenatal and postpartum depression, which may in turn put infants at risk for physical and mental health difficulties [3, 7, 8]. In order to mitigate risk, pre- and postpartum mental health screening are imperative, particularly for women where a known trauma history is identified. Trauma-informed approaches to patient care, as well as increased support during and after pregnancy, are warranted to offset the relative risk of ACEs on maternal mental health.

## Supplementary Information

**Additional file 1.**

## Data Availability

The data used for this meta-analysis are publicly available in the research studies. The full dataset can be requested from the corresponding author on reasonable request.

## Abbreviations

ACEs: Adverse Childhood Experiences
