# Supplementary material for: Adverse childhood experiences and maternal anxiety and depression: a meta-analysis
Source: BMC Psychiatry. 2021 Jan 11;21:28. doi: 10.1186/s12888-020-03017-w (PMC7802164; doi:10.1186/s12888-020-03017-w)
Supplement: Supplementary file 1 — Additional file 1. [file 12888_2020_3017_MOESM1_ESM.docx]

**Supporting Information 1**

*PRISMA Checklist*

**
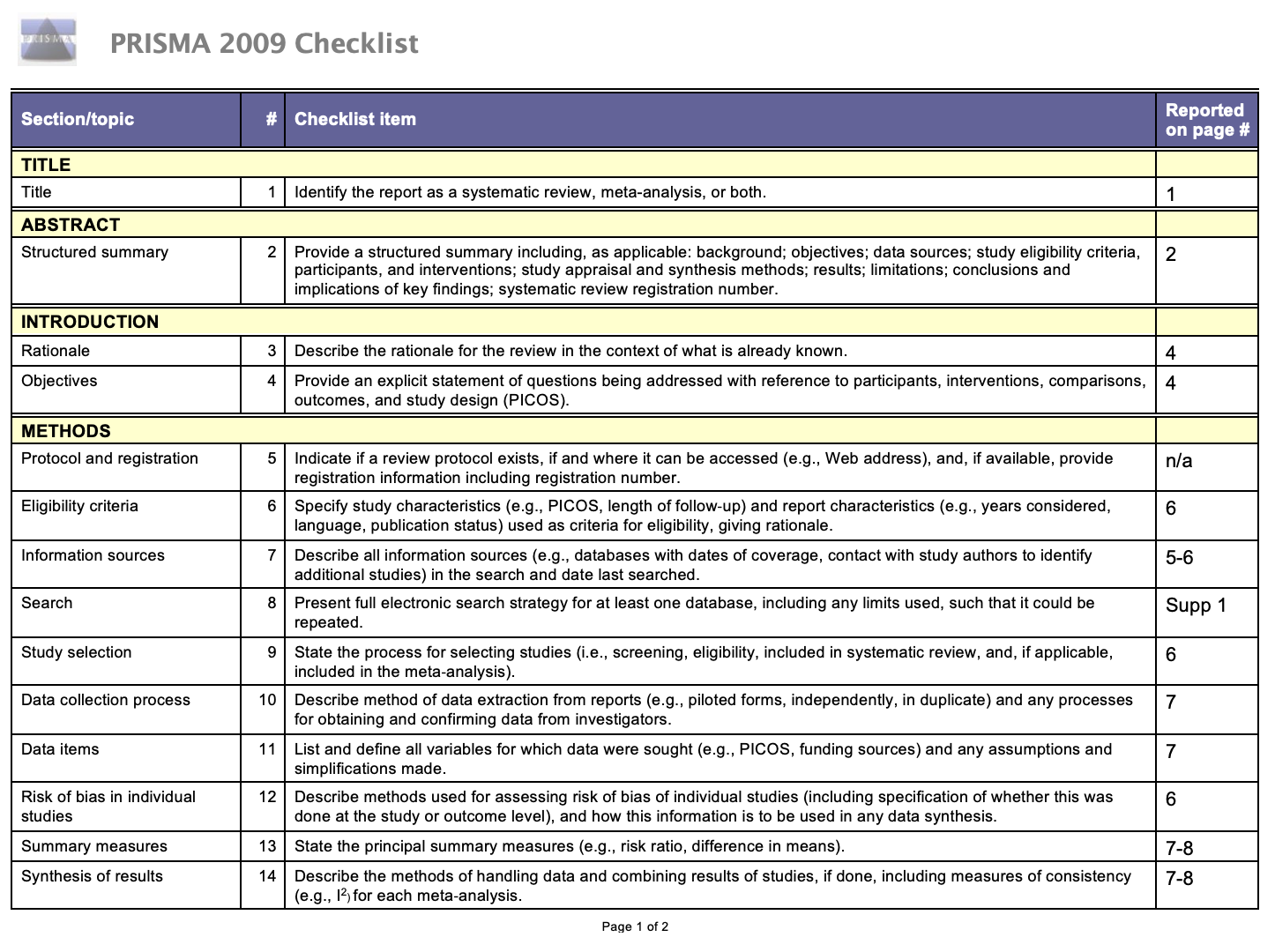
**

**
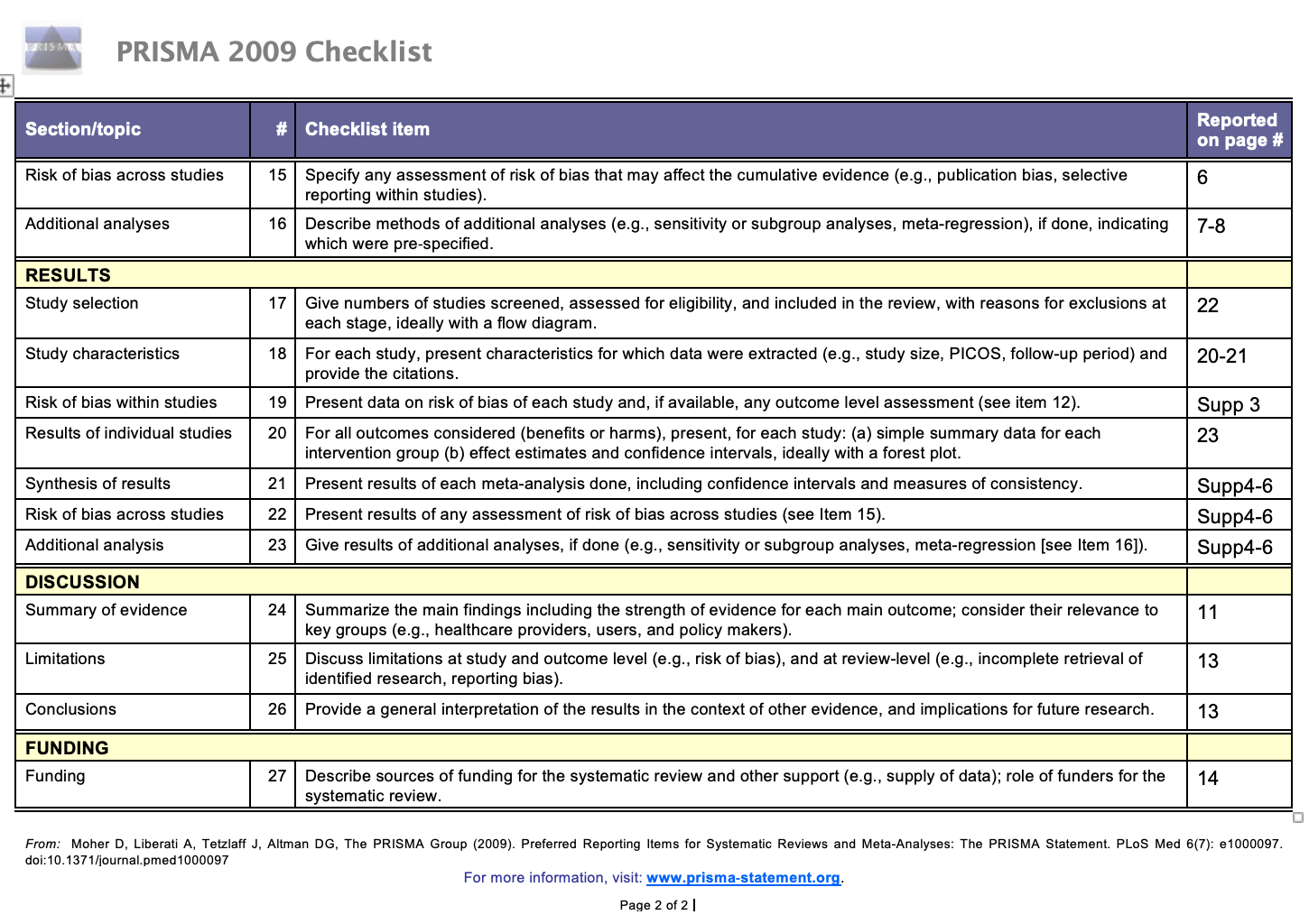
**

**Supporting Information 2**

*Example Search Strategy from PsycINFO*

| Database: PsycINFO <1806 to November 2018>  Search Strategy: |
| --- |
| 1     (adverse adj2 child* adj2 (experience* or event*)).mp. (1485)  2     aces.mp. (610)  3     1 or 2 (1677)  4     limit 3 to yr="1998 -Current" (1596) |

Note. *Indicates that words were truncated it order instruct the database to capture all variant

**Supporting Information 3**

*Study Quality Evaluation Criteria*

| **Study Quality Evaluation**^[[1]](#footnote-1)^ | | |
| --- | --- | --- |
| Item | Question | Coding |
| 1. Question | Was the research question or objective in this paper clearly stated? | 0-No  1-Yes |
| 1. Population | Was the study population clearly specified and defined? | 0-No  1-Yes |
| 1. Participation | Was the participation rate of eligible persons at least 50%? | 0-No  1-Yes |
| 1. Inclusion/Exclusion Criteria | Were all the subjects selected or recruited from the same or similar populations (including the same time period)? Were inclusion and exclusion criteria for being in the study prespecified and applied uniformly to all participants? | 0-No  1-Yes |
| 1. Sample Size | Was a sample size justification, power description, or variance and effect estimates provided? | 0-No  1-Yes |
| 1. Exposures | For the analyses in this paper, were the exposure(s) of interest measured prior to the outcome(s) being measured? | 0-No  1-Yes |
| 1. Timeframe | Was the timeframe sufficient so that one could reasonably expect to see an association between exposure and outcome if it existed? | 0-No  1-Yes |
| 1. Levels of Exposure | For exposures that can vary in amount or level, did the study examine different levels of the exposure as related to the outcome (e.g., categories of exposure, or exposure measured as continuous variable)? | 0-No  1-Yes |
| 1. Independent Variable | Were the exposure measures (independent variables) clearly defined, valid, reliable, and implemented consistently across all study participants? | 0-No  1-Yes |
| 1. Longitudinal/Repeated ACEs | Was the exposure(s) assessed more than once over time? | 0-No  1-Yes |
| 1. Dependent Variable | Were the outcome measures (dependent variables) clearly defined, valid, reliable, and implemented consistently across all study participants? | 0-No  1-Yes |
| 1. Objectivity independent variable | Does the study use objective reports or multiple-methods to measure maternal ACEs?  Objective measure = child abuse reports  Multiple methods = self-report and corroborated reports. | 0-self report  1-objective measure/multiple methods |
| 1. Objective dependent variables | Does the study use different reporters or multiple-methods to measure maternal health/mental health outcomes?  Objective measure = hospital report, diagnosis by physician, measurement by health care professional | 0-self report  1-objective measure/multiple methods |
| 1. Lost to Follow-Up | Was loss to follow-up after baseline 20% or less? | 0-No  1-Yes |
| 1. Confounder | Were key potential confounding variables measured and adjusted statistically for their impact on the relationship between exposure(s) and outcome(s)? | 0-No  1-Yes |
| **Total** | A sum of all items was calculated to obtain a total quality score (0-15). |  |

**Supporting Information 4**

| First Author, Year, Reference | Research Question | Defined Sample | Participation | Inclusion/Exclusion Criteria | Sample Size | Exposures | Timeframe | Levels of Exposure | Independent Variable | Repeated ACEs | Dependent Variable | Objective Measure (Maternal ACEs) | Objective Measure (Depression/Anxiety) | Lost to Follow-Up | Confounders | Total Score (0-15) |
| --- | --- | --- | --- | --- | --- | --- | --- | --- | --- | --- | --- | --- | --- | --- | --- | --- |
| Angerud 2018 | Yes | Yes | Yes | Yes | No | No | Yes | Yes | Yes | No | Yes | No | No | No | No | 8 |
| Appleton 2019 | Yes | Yes | Yes | Yes | No | No | Yes | No | Yes | No | Yes | No | Yes | Yes | No | 9 |
| Folger 2018 | Yes | Yes | Yes | Yes | No | No | Yes | Yes | Yes | No | Yes | No | No | No | No | 8 |
| Fredriksen 2017 | Yes | Yes | Yes | Yes | No | No | Yes | Yes | Yes | No | Yes | No | No | No | No | 8 |
| Hantsoo 2019a | Yes | No | Yes | Yes | Yes | No | Yes | No | Yes | No | Yes | No | No | Yes | Yes | 9 |
| Howell 2017 | Yes | No | Yes | No | No | No | Yes | Yes | Yes | No | Yes | No | No | Yes | No | 7 |
| Letourneau 2019 | Yes | Yes | Yes | Yes | No | No | Yes | Yes | Yes | No | Yes | No | No | Yes | No | 9 |
| Menke 2018 | Yes | Yes | Yes | Yes | No | Yes | Yes | Yes | Yes | No | Yes | No | No | Yes | Yes | 11 |
| Mersky 2018 | Yes | Yes | Yes | Yes | No | Yes | Yes | Yes | Yes | Yes | Yes | No | No | Yes | Yes | 12 |
| Miller-Graff 2018 | Yes | Yes | Yes | Yes | No | Yes | Yes | Yes | Yes | No | Yes | No | No | Yes | Yes | 11 |
| Morrison 2017 | Yes | Yes | Yes | Yes | No | No | Yes | No | Yes | No | Yes | No | No | Yes | No | 8 |
| Narayan 2018 | Yes | Yes | Yes | Yes | No | No | Yes | Yes | Yes | No | Yes | No | No | Yes | No | 9 |
| Racine 2020 | Yes | Yes | Yes | Yes | No | No | Yes | No | Yes | No | Yes | No | No | No | Yes | 8 |
| Wajid 2019 | Yes | Yes | Yes | Yes | No | No | Yes | No | Yes | No | Yes | No | No | Yes | No | 8 |
| Young-Wolff 2019 | Yes | Yes | Yes | Yes | No | No | Yes | Yes | Yes | No | Yes | No | Yes | Yes | Yes | 11 |

*Quality Assessment of Studies Included*

**Supporting Information 5**

Funnel plot for association between maternal ACEs and prenatal depression.


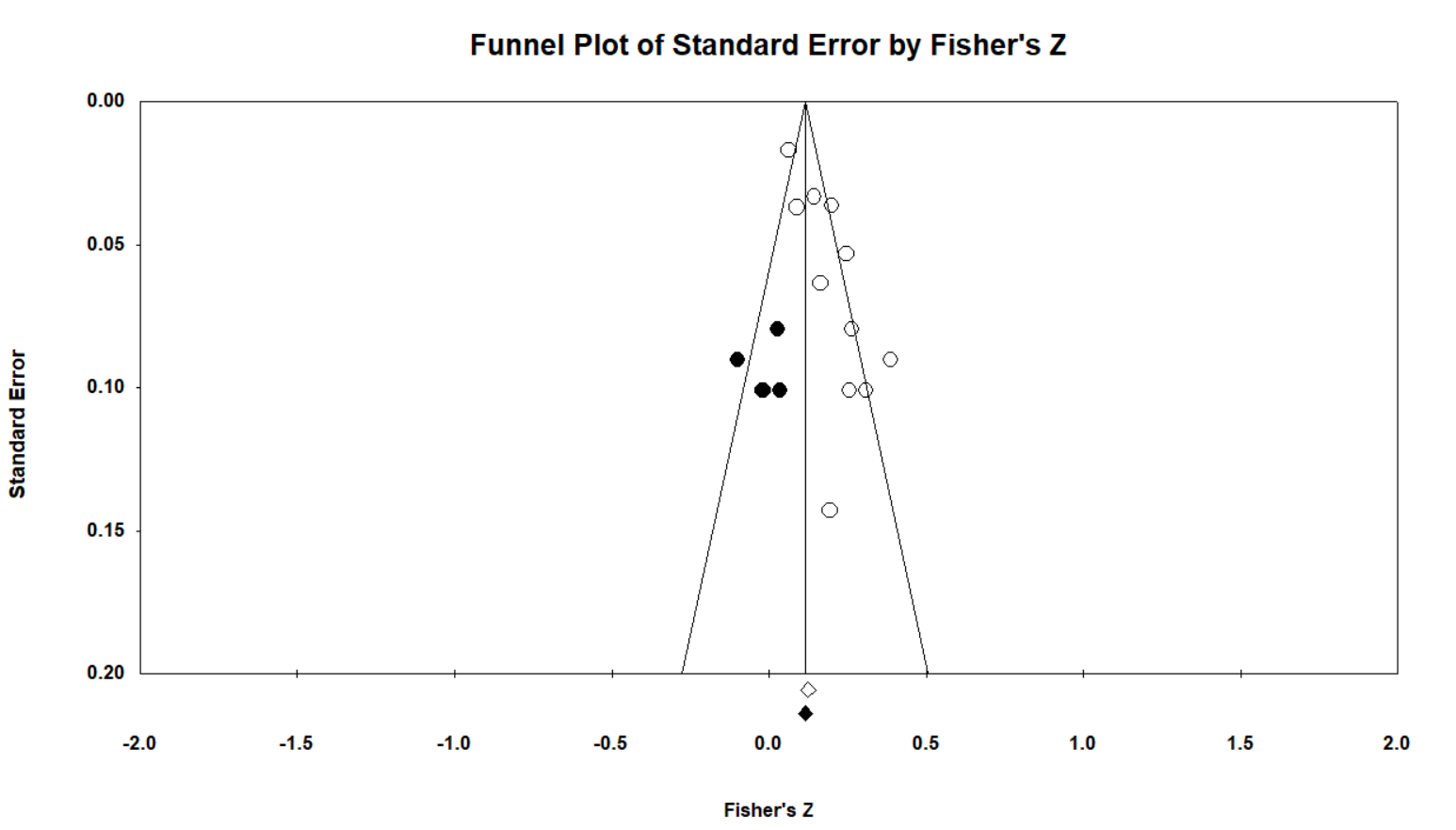


Legend: The funnel plot is a measure of the study size (y-axis) as a function of the effect size (x-axis). Dark circles indicated imputed studies when asymmetry is detected, while white circles indicate observed studies. The mean prevalence estimate is indicated by the middle vertical line and the contour lines represent the region in which 95% of observed studies should lie if there were no publication bias. The white diamond represents the observed mean effect size, and the black diamond represented the adjusted mean effect size. Studies with large sample sizes appear at the top of the graph, whereas studies with smaller effect sizes appear at the bottom of the graph.

**Supporting Information 6**

*Results of Moderator Analyses for the Association Between Maternal ACEs and Prenatal Depression*

| **Prenatal Depression** |  |  |  |  |  |
| --- | --- | --- | --- | --- | --- |
| **Categorial Moderators**^a^ | **k** | ***r*** | **95% CI** | **Homogeneity *Q*** | ***p*** |
| Sociodemographic risk |  |  |  | 2.714 | .10 |
| No risk | 3 | .13 | .047, .213 |  |  |
| At-risk sample | 9 | .22 | .152, .288 |  |  |
| **Continuous Moderators** | **k** | ***b*** | **95% CI** | ***Z*** | ***p*** |
| Maternal age at outcome | 10 | -.002 | -.02, .024 | -.16 | .872 |
| Prenatal timing of outcome | 9 | -.002 | -.021, .016 | -.23 | .819 |
| Study Quality | 12 | -.018 | -.06, .019 | -.96 | .336 |

Note. k=number of studies; *b*=estimate; r=correlation coefficient, CI=confidence interval.

^a^The categorical moderator, syndromal level of depression, was not analysed as there fewer than three studies in one of the categories.

**Supporting Information 7**

Funnel plot for association between maternal ACEs and postpartum depression.


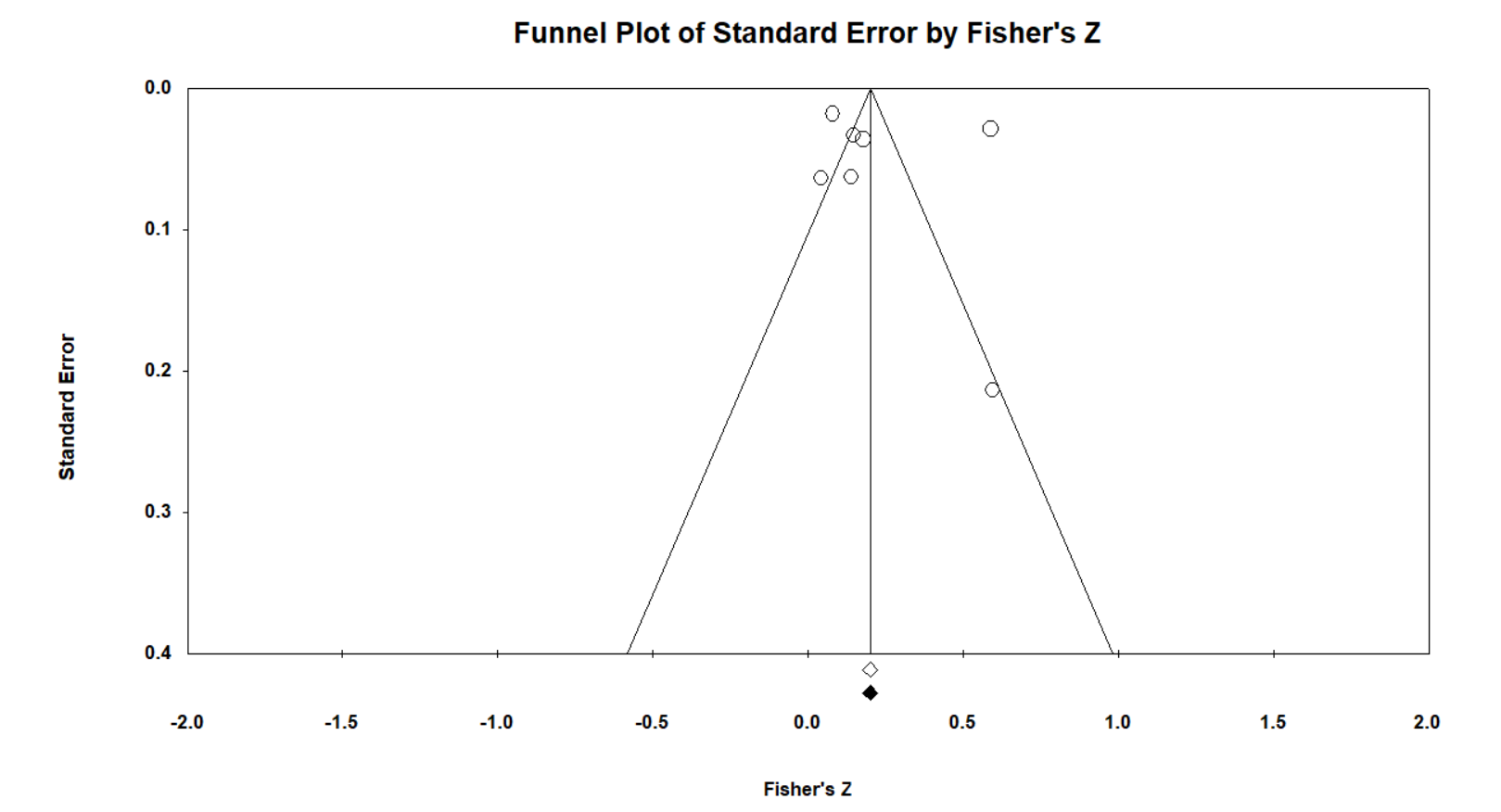


Legend: The funnel plot is a measure of the study size (y-axis) as a function of the effect size (x-axis). Dark circles indicated imputed studies when asymmetry is detected, while white circles indicate observed studies. The mean prevalence estimate is indicated by the middle vertical line and the contour lines represent the region in which 95% of observed studies should lie if there were no publication bias. The white diamond represents the observed mean effect size, and the black diamond represented the adjusted mean effect size. Studies with large sample sizes appear at the top of the graph, whereas studies with smaller effect sizes appear at the bottom of the graph.

**Supporting Information 8**

*Results of Moderator Analyses for the Association Between Maternal ACEs and Postpartum Depression*

| **Postnatal Depression** |  |  |  |  |  |
| --- | --- | --- | --- | --- | --- |
| **Categorial Moderators^a^** | **k** | ***r*** | **95% CI** | **Homogeneity *Q*** | ***p*** |
| Sociodemographic risk |  |  |  | 1.27 | .26 |
| No risk | 3 | .13 | .07, .19 |  |  |
| At-risk sample | 4 | .32 | -.009, .579 |  |  |
| **Continuous Moderators** | **k** | ***b*** | **95% CI** | ***Z*** | ***p*** |
| Maternal age at outcome | 5 | -.087 | -.244, .071 | -1.08 | .28 |
| Postnatal timing of outcome | 6 | .041 | .007, .075 | 2.38 | .02 |
| Study Quality | 7 | -.072 | -.21, .07 | -1.03 | .30 |

Note. k=number of studies; *b*=estimate; r=correlation coefficient, CI=confidence interval.

^a^The categorical moderator, syndromal level of depression, was not analysed as there fewer than three studies in one of the categories.

**Supporting Information 9**

Funnel plot for association between maternal ACEs and prenatal anxiety.


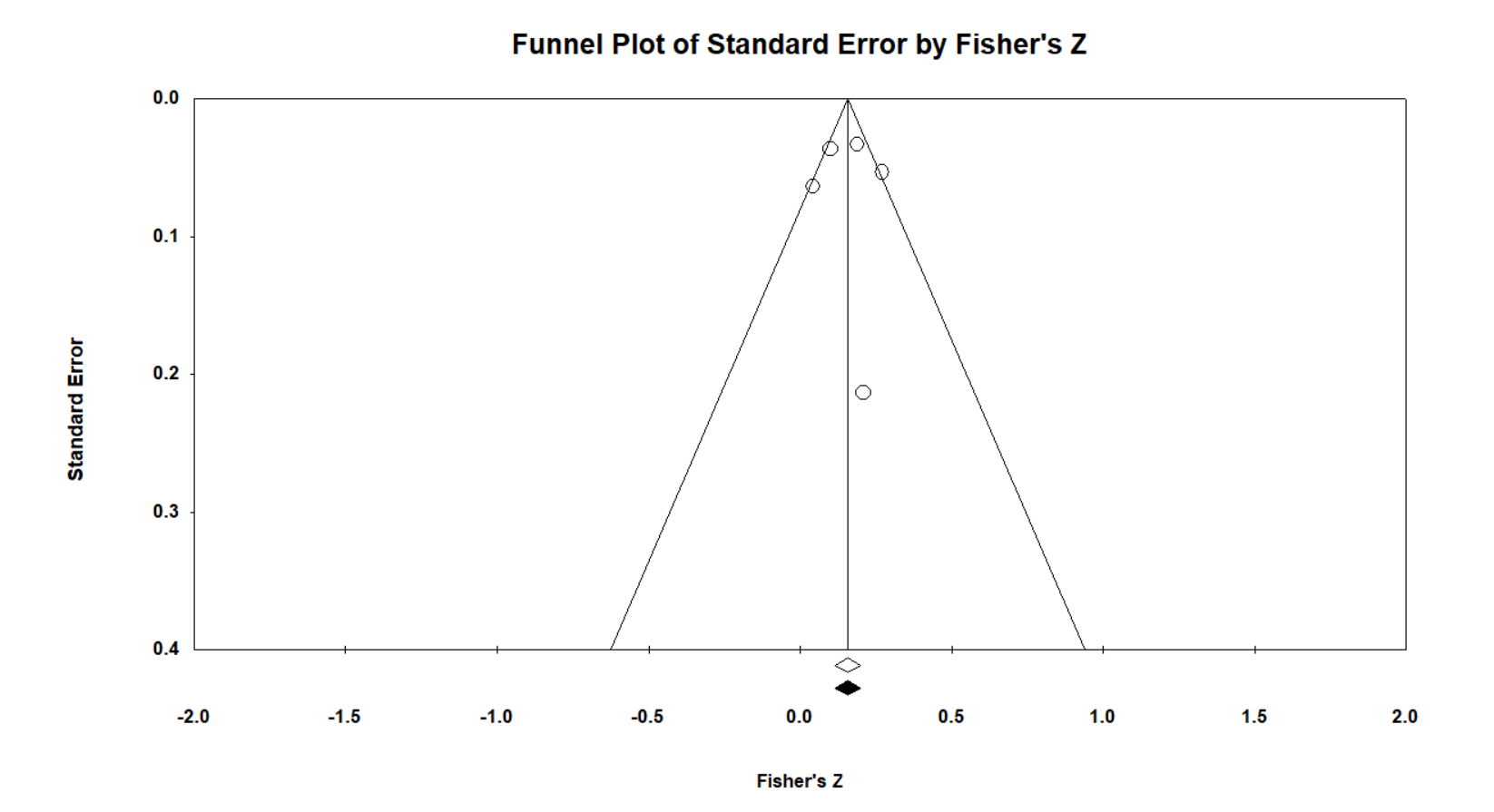


Legend: The funnel plot is a measure of the study size (y-axis) as a function of the effect size (x-axis). Dark circles indicated imputed studies when asymmetry is detected, while white circles indicate observed studies. The mean prevalence estimate is indicated by the middle vertical line and the contour lines represent the region in which 95% of observed studies should lie if there were no publication bias. The white diamond represents the observed mean effect size, and the black diamond represented the adjusted mean effect size. Studies with large sample sizes appear at the top of the graph, whereas studies with smaller effect sizes appear at the bottom of the graph.

**Supporting Information 10**

*Results of Moderator Analyses for the Association Between Maternal ACEs and Prenatal Anxiety*

| **Prenatal Anxiety^a^** |  |  |  |  |  |
| --- | --- | --- | --- | --- | --- |
| **Continuous Moderators** | **k** | ***b*** | **95% CI** | ***Z*** | ***p*** |
| Maternal age at outcome | 4 | -.006 | -.10, .09 | -.12 | .904 |
| Prenatal timing of outcome | 4 | -.060 | -.108, -.013 | -2.47 | .014 |
| Study Quality | 5 | -.0004 | -.06, .06 | -.01 | .99 |

Note. k=number of studies; *b*=estimate; r=correlation coefficient, CI=confidence interval.

**^a^**No categorical moderators were included (i.e., syndromal level or sociodemographic risk) due to k<3 for subgroups of these variables.

1. Adapted from the NIH Quality Assessment Tool for Observational Cohort and Cross-Sectional Studies to evaluate any bias in the study or measurements. This measure is available at https://www.nhlbi.nih.gov/health-topics/study-quality-assessment-tools [↑](#footnote-ref-1)
